# Double the Double: Revisiting BCL11B's Multimerization

**DOI:** 10.1002/prot.26811

**Published:** 2025-02-20

**Authors:** Anne Susemihl, Norman Geist, Piotr Grabarczyk, Christian A. Schmidt, Mihaela Delcea, Lukas Schulig

**Affiliations:** ^1^ Department of Biophysical Chemistry, Institute of Biochemistry University of Greifswald Greifswald Germany; ^2^ Comprehensive Cancer Center Mecklenburg‐Vorpommern University of Greifswald Greifswald Germany; ^3^ Department of Pharmaceutical and Medicinal Chemistry, Institute of Pharmacy University of Greifswald Greifswald Germany

**Keywords:** BCL11B, CCHC zinc finger, crosslinking, protein–protein docking, protein–protein interactions, replica‐exchange molecular dynamics, size exclusion chromatography, tetramers, TIGER2h^PE^

## Abstract

The transcription factor B Cell Lymphoma/Leukemia 11B (BCL11B) exerts a bi‐directional function in cancer, with its role as an emerging therapeutic target in cancer treatment being particularly intriguing. *BCL11B* knockouts in cultured T cells revealed the acquisition of properties characteristic of natural killer cells, hinting at its importance in innate versus adaptive immune regulation. Our previous studies using Förster Resonance Energy Transfer‐assisted Fluorescence‐Activated Cell Sorting and Hybrid Solvent Replica‐Exchange Simulations indicated that BCL11B forms dimers, with this being a prerequisite for its activity. However, size exclusion chromatography and crosslinking experiments have challenged this view, suggesting that BCL11B forms tetramers instead. An atypical CCHC zinc finger motif within the *N*‐terminal region of the protein mediates multimerization and a novel 3D structure is presented based on extensive replica‐exchange simulations in strong agreement with experimental data. The physiological relevance of multimer formation of this zinc finger protein has been demonstrated previously. Therefore, understanding the nature of BCL11B's multimerization could potentially enhance our ability to target this protein effectively, hopefully paving the way for novel BCL11B‐targeted therapies.

## 1 Introduction

The transcription factor B Cell Lymphoma/Leukemia 11 B (BCL11B) plays a fundamental role in T cell development and maintenance of T cell identity [1, 2]. It is a member of the Krüppel‐like family and comprises six C2H2 zinc finger domains localized in Exon 4 [3]. The *N*‐terminal region associates with the Nucleosome Remodeling and Deacetylase (NuRD) complex [4] and contains an additional, atypical CCHC zinc finger (ZF0) responsible for multimerization, necessary and sufficient for the repressor activity of the BCL11B/NuRD complex [5].

Because of its importance for the differentiation of a multitude of tissues and organs, the development of many of them, like the central nervous system and T cells, is severely disturbed in the absence of BCL11B. Further studies with tissue‐specific knockout mouse models showed drastic skin, teeth, and mammary gland abnormalities. A dysregulation in *BCL11B* expression and genetic aberrations involving the *BCL11B* gene are associated with a plethora of pathologies, such as leukemia, cancer, and neurodegenerative disorders [6].

Depletion of the *BCL11B* gene in mature mouse and human T cells was shown to induce an innate, Natural Killer‐like transcription program that converted T cells into NK/T cell chimeras with unique properties and superior cytotoxicity against tumor cells [7, 8]. BCL11B, as a transcriptional regulator, can act as both an activator and a repressor [7]. The bi‐directional role [9] of BCL11B becomes relevant in the context of T cell acute lymphoblastic leukemia (T‐ALL). In 10%–16% of this hematologic malignancy, mutations in BCL11B modifying its DNA binding properties were found, whereas 10% of T‐ALL cases were characterized by elevated protein levels [10]. Overexpression of BCL11B was also found to generate apoptosis resistance, being chaperoned by chemo‐resistance based on T‐ALL cells accumulating in G1 phases [11].

The first identified germline de novo mutation within *BCL11B* was characterized by the patient lacking T cells, suffering from multiple organ defects, and mental retardation [12]. The N441K mutation was present in one of the *BCL11B* alleles but mimicked the “null” phenotype. That suggested that BCL11B functions as a dimer and is unable to interact with and regulate its target genomic regions in the presence of a defective variant.

The molecular assembly of BCL11B into dimers via the ZF0 was previously postulated based on the Förster Resonance Energy Transfer‐assisted Fluorescence‐Activated Cell Sorting (FRET‐FACS) assay, which utilized ECFP and EYFP as FRET pair. The structure of the zinc finger domain and a critical monomer–monomer interface were elucidated using hybrid solvent replica exchange molecular dynamics simulations (TIGER2h(s)) [13] and mutagenesis experiments. The formation of the dimers was mainly mediated through hydrophobic interactions at this interface [14].

In this study, we report an updated model of BCL11B multimerization and propose its primary form as a highly symmetrical tetramer. The 3D structure of the multimeric ZF0 domain assembly of BCL11B was obtained through extensive atomistic simulations, leveraging the TIGER2h^PE^ replica exchange algorithm [15]. This was complemented by analytical size exclusion chromatography (SEC), crosslinking experiments, circular dichroism spectroscopy, and reinterpretation of the FRET‐FACS data from our previous studies. It has been proven that multimerization of BCL11B is crucial for its activity and therefore represents a promising target for the inhibition of BCL11B. The updated model presented here provides new insights into the biology of BCL11B and opens new opportunities to drug this by now undruggable transcription factor, offering therapeutic interventions against various conditions.

## 2 Results and Discussion

**FIGURE 1:**
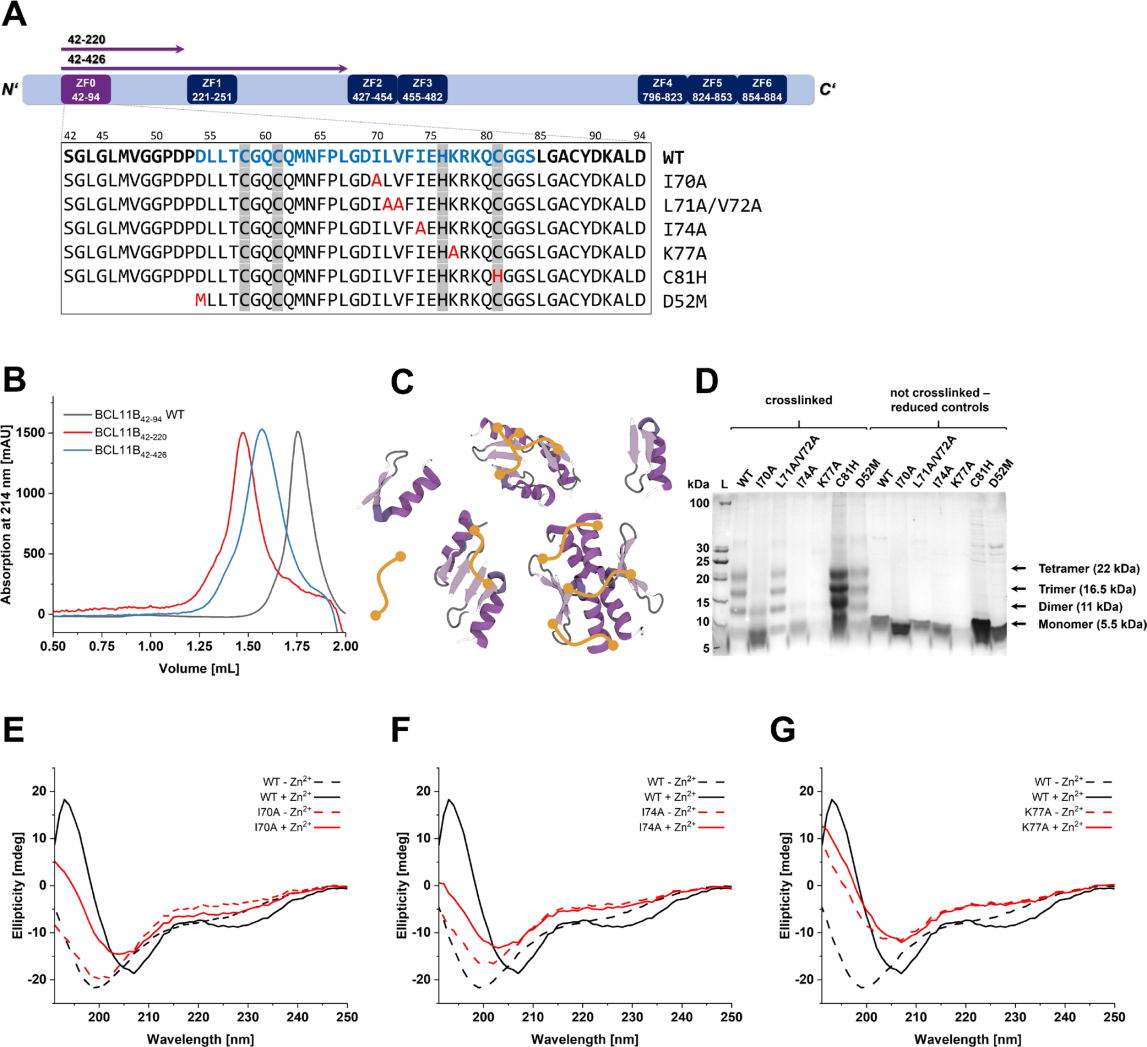
Characterization of the multimerization states and secondary structures of different BCL11B species. (A) Sequence overview of different BCL11B expression constructs and mutants (red) deduced from FRET‐FACS experiments. The sequence highlighted in blue denotes the shortest possible construct to form a stable ZF0 fold and was used during MD simulations. Zinc binding residues are shown in gray. (B) Elution profiles from size exclusion chromatography (SEC) of BCL11B_42–94_ WT (gray), BCL11B_42–220_ WT (red), and BCL11B_42–426_ WT (blue). The elution volumes of 1.79 mL (WT), 1.47 mL (220), and 1.57 mL (426) each correspond to calculated stoichiometric factors of 7.0 (WT), 16.6 (220), and 4.0 (426). As SEC data are suitable for a rough estimation of molecular weights and all three constructs appeared as larger complexes than the expected dimers, we continued with crosslinking experiments. (C) Schematic illustration of crosslinking (orange) nearby subunits (cartoon) within higher‐order complexes. (D) Reducing 16% tricine SDS‐PAGE with crosslinked and control samples of BCL11B_42–94_ WT and mutants. Circular dichroism spectra of BCL11B_42–94_ WT in comparison to the mutants (E) BCL11B_42–94_ I70A, (F) BCL11B_42–94_ I74A, and (G) BCL11B_42–94_ K77A, with Zn^2+^ ions removed or added, respectively.

We aimed to follow up on characterizing BCL11B multimers of the recombinantly expressed protein. In addition to the *N*‐terminal wild‐type (WT) zinc finger domain BCL11B_42–94_ (ZF0) and various mutants, we expressed C‐terminally prolonged constructs (Figure 1A). In BCL11B_42–220_, the amino acid sequence is extended up until the first C2H2 zinc finger domain (ZF1), while for BCL11B_42–426_, ZF1 is included and also the presumably disordered region until the next zinc finger domain (ZF2).

During the purification of the recombinantly expressed BCL11B_42–94_ WT and the longer constructs (BCL11B_42–220_, BCL11B_42–426_) from *E. coli*, it appeared that the ZF0 domain forms larger complexes than anticipated. Size exclusion chromatography (Figure 1B) suggests (upon using the calibration curve shown in Figure S1) the formation of tetramers for BCL11B_42–426_ and apparent larger complexes for BCL11B_42–94_ and BCL11B_42–220_. As the running behavior of the SEC column is very dependent on the shape of the protein, we believe that the shorter protein constructs exhibit a different behavior and elute faster than the larger and more disordered BCL11B_42–426_ construct, hence resulting in the indication of even larger complexes. Mutants of the short construct (BCL11B_42–94_) were also analyzed (Figure S2). The WT, C81H, L71A/V72A, and D52M eluted at volumes corresponding to larger complexes, whereas the mutants I70A, I74A, and K77A suggested the formation of smaller complexes.

Crosslinking experiments were carried out to obtain further insights into the level of multimerization for the *N*‐terminal domain (ZF0). The BCL11B constructs were incubated with bis‐succinimide ester‐activated PEG (BS(PEG)5) linker of 21.7 Å in length. The BS(PEG)5 crosslinkers form covalent amide bonds between primary amines (lysines, *N*‐terminus) and, in our case, would stably connect BCL11B monomers within larger multimeric species, that is, dimers, trimers, tetramers, and so on. (Figure 1C). In this way, multimers are not denatured to monomers in gel electrophoresis, allowing the observation of all intermediate and stable assemblies.

BCL11B_42–94_ WT exhibits four bands at heights corresponding to monomers, dimers, trimers, and tetramers, with no species larger than tetramers apparent (Figure 1D). The same applies to the slightly shorter mutant D52M, the double mutant L71A/V72A, and C81H. Both isoleucine mutants I70A and I74A, as well as the lysine mutant K77A, showed only two bands, indicating dimer formation at max. Crosslinking experiments with the longer constructs BCL11B_42–220_ and BCL11B_42–426_ were also carried out but did not result in the expected display of multimers (data not shown). A plausible reason is the shielding of the core tetramer assembly by large disordered regions surrounding it, rendering its lysine residues inaccessible. Instead, the crosslinkers react with neighboring lysine residues within the disordered regions of one monomer that follow beyond ZF0.

Furthermore, we examined the secondary structures of the zinc finger domains each in the presence and in the absence of Zn^2+^ ions (Figure 1E–G). As presented in our previous study [16], BCL11B_42–94_ WT exhibits the typical ββα‐fold for zinc finger domains [17]. In contrast, the BCL11B_42–94_ mutants I70A, I74A, and K77A rather resemble the fold of the zinc‐deprived WT, indicating an incomplete zinc finger fold.

**FIGURE 2:**
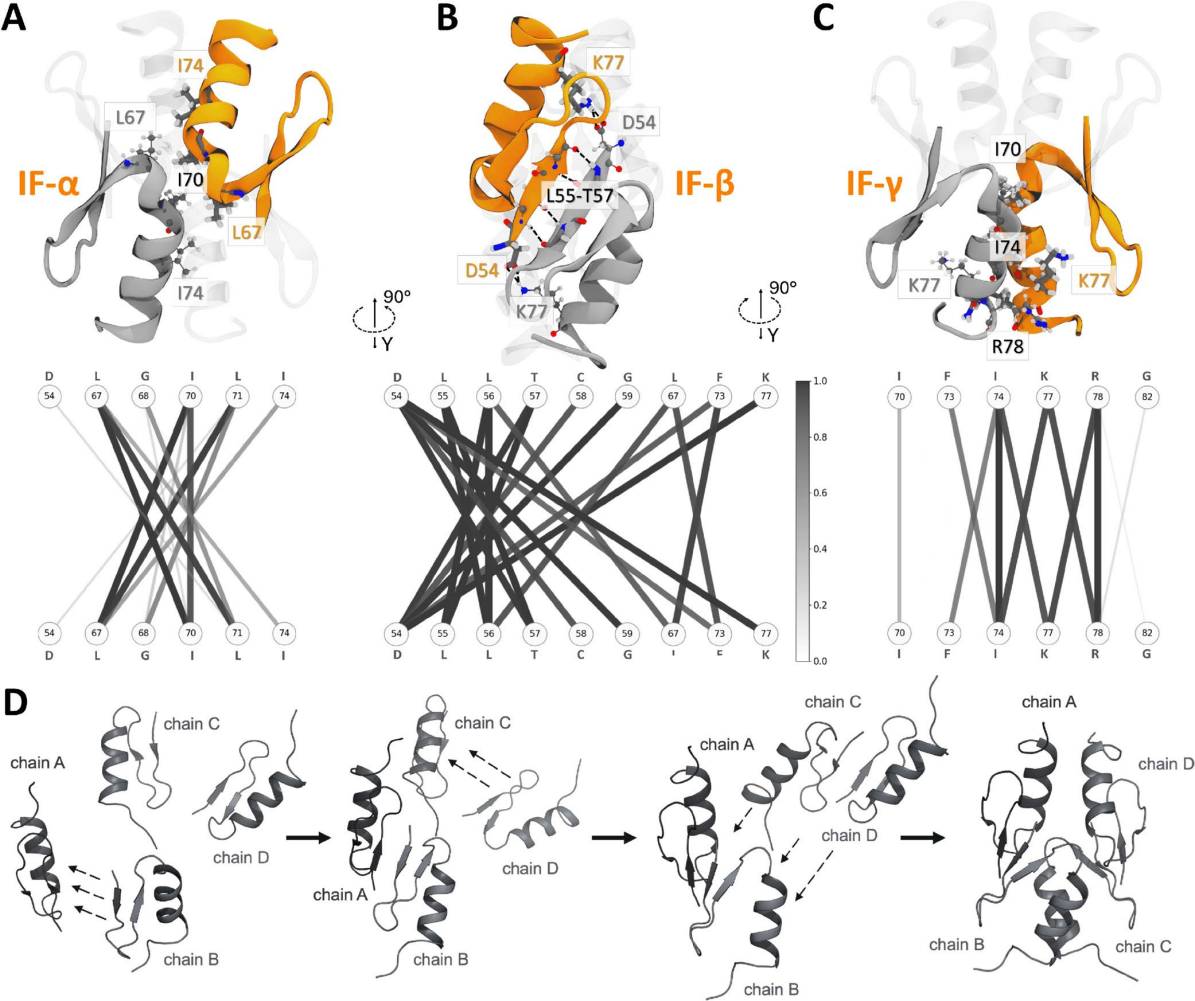
(A–C) Tetrameric structure of the BCL11B_54–82_ ZF0 CCHC zinc finger domain in cartoon style, highlighting three different core interfaces, including (A) the previously proposed dimer interface (IF‐α), (B) the new major β‐dimer building block interface (IF‐β), stacking the β‐sheets of two monomers and stabilized by a salt‐bridge K77↔D54, and (C) an additional interface between α‐helical regions of two neighboring chains between two β‐dimers (IF‐γ). (bottom) Corresponding contact probability network for each interface. Nodes display residue IDs labeled with their corresponding one‐letter amino acid codes. The thickness and shade of the connecting lines indicate the proportion of time each contact persisted within the main contact‐contact principal component analysis (ccPCA) cluster of simulation R2 (Table S1). (D) Schematic illustration of the presumed formation of the BCL11B ZF0 tetramer from four monomeric zinc finger motifs involves multiple stages. Initially, dimers are assembled by joining the sheets of residues D54 to T57 (IF‐β) between monomers, building a β‐dimer. Next, two β‐dimers form a tetramer as a “dimer of dimers” via two emerging hydrophobic interfaces: IF‐α, described in our earlier work and an additional interface IF‐γ [14].

To investigate a tetrameric structure of the BCL11B ZF0 domain, we employed the improved TIGER2h^PE^ method to sample the assembly within a system comprising four monomers, adhering to the methodology described in our previous study [14]. In brief, four ZF0 constructs (BCL11B_54–82_), containing only the basic zinc finger fold, were randomly oriented in close proximity and sampled twice using varying temperature settings to boost convergence (Figure 2A, Table S1).

During the first simulation (R1), it quickly became apparent that a novel predominant dimer structure emerged, wherein two monomers connect across the β‐sheet around residues D55 to T57 (Figure 2B), in the following referred to as β‐dimer. This dimer formation occurred rapidly during our simulation and remained stable over extended periods. Furthermore, we observed the assembly of these dimers into various tetrameric assemblies, often involving the previously identified monomer‐monomer interface from our preceding work (Figure 2A), in the following referred to as IF‐α. The new β‐dimer structure was not observed in our previous work investigating an assumed dimer formation since a longer sequence was utilized during simulations. There, when we first examined the monomeric fold, an additional α‐helical region at the *N*‐terminal was attached to this position on the β‐sheet. Since the monomers were then held rigid during dimer formation simulations, this interface was obstructed, rendering the predominant dimer structure inaccessible at that time and shifting the conformational search to a different interface.

We applied a contact–contact principal component analysis (ccPCA) to identify the most common structural clusters from the initial simulation data collected via TIGER2h^PE^ during R1, revealing various structural hot spots (Figure S3). Several clusters showed the formation of the β‐dimer interacting with other monomers in various configurations. Additionally, some clusters displayed the assembly of a dimer across the IF‐α interface and a β‐dimer in a rotated fashion, also forming a stable hydrophobic core. However, near the end of R1, one cluster emerged, showing two β‐dimers in a sandwich‐like configuration, resulting in a highly symmetrical structure. Interestingly, this configuration led to the re‐emergence of the previously found IF‐α interface, just from the assembly of these two β‐dimers (Figure 2A).

To determine the most stable tetrameric structure, we repeated the sampling with TIGER2h^PE^ at a higher maximum temperature (R2) to enhance the likelihood of escaping intermediate structures and converge closely to the Boltzmann distribution of states. Using ccPCA analysis again, we found that only one structural motif persisted, namely the assembly of two β‐dimers (Figure S4). For validation, we compared our structure with predictions from the very recent AlphaFold 3 [18] and obtained the very same structure in all models with low average RMSD values between 1.15 and 2.61 Å (Figure S5).

We then analyzed the contacts between the monomers in this predominant cluster from R2 and formed contact networks (Figure 2, middle row) to be compared with the FRET‐FACS experiments from our previous work [14].

From our analysis, we derive a formation process of the BCL11B tetramers (Figure 2D) that is characterized by three different interfaces (Figure 2A–C). First, β‐dimers build rapidly from available monomers via the IF‐β interface (Figure 2B). The major interaction within IF‐β appears symmetrically across the β‐sheet: D54↔T57, D54↔C58, D54↔G59, L55↔L56, L55↔T57, and L56↔L56, including additional interactions between L67↔L56, L67↔F73, and F73↔D54. A charge‐lock between D54 and K77 stabilizes the complex further. Next, these β‐dimers will assemble into symmetrical tetramers involving the IF‐α interface (Figure 2A), including multiple hydrophobic interactions occurring symmetrically: that is, L67↔I70, L67↔L71, and L67↔I74. In addition, an interface IF‐γ emerges between α‐helical regions of neighboring monomers from two different β‐dimers (Figure 2C), again building symmetrical hydrophobic contacts: I74↔F73, I74↔I74, I74↔K77, K77↔R78, and R78↔R78. Each of these interfaces exists twice in one tetramer, resulting in a highly stable complex, as seen during R2. Once found, this structure was never lost.

Based on simulation and experimental data, several residues were identified that either had a significant impact on the multimerization of the ZF0 domain as a tetramer or did not interfere.

The zinc finger mutant C81H, which modifies the atypical CCHC zinc finger domain to a common C2H2 zinc finger domain, showed results similar to the WT. Previous FRET‐FACS results indicated the formation of at least dimers, and crosslinking experiments now confirmed tetramer formation. This mutation did not impact the multimerization state of ZF0, as the typical zinc finger fold remained intact.

The *N*‐terminally shortened construct D52M, which lacks residues immediately preceding the β‐sheet, did not alter the multimerization state. This indicates that these residues are not directly involved in the interfaces and that the classical zinc finger motif is the only required structural entity. This is also in agreement with our simulations, where the respective *N*‐terminal amino acids were absent and not required for the formation of tetramers.

The double mutant L71A/V72A continued to form larger complexes, which were identified as tetramers in crosslinking experiments (Figure 1D) and are also consistent with the earlier FRET‐FACS data. The residue L71 interacts with L67 in the IF‐α interface between two β‐dimers. Although this mutation may influence the stability of the protein–protein interface, it does not disrupt dimer‐dimer formation. V72, being more solvent‐exposed, does not appear to be directly involved in binding.

Both mutants I70A and I74A, located within the hydrophobic interfaces between two β‐dimers, resulted in the formation of dimers only (Figure 1D), indicating the importance of these residues in direct binding during the “dimer of dimers” formation. The contact analysis underscored their role in IF‐α and IF‐γ. I70 and I74 interact with L67 within the IF‐α interface of two β‐dimers, but their mutations may disrupt interactions at IF‐γ (Figure 2C), causing complex degradation. However, CD spectra (Figure 1E,F) also indicate altered folding of the zinc finger domains for both mutants, with clear distinction to the typical ββα‐fold of the BCL11B_42–94_ WT. Therefore, degradation of the tetrameric complex might also be caused by the altered secondary structure. Previous FRET‐FACS data indicated the presence of monomers, but because of the qualitative nature of the assay, dimer formation was likely misinterpreted as monomerization, due to the resulting lower signal.

The lysine mutation K77A plays a decisive role in zinc complexation by favoring the deprotonation of thiol groups [19]. This importance is also highlighted by the CD spectra (Figure 1G), which do not show the typical zinc finger fold, neither with or without Zn^2+^ ions. From our simulation data, it could also be shown that K77 forms a salt bridge with D54 and thus stabilizes the β‐dimer via a charge‐lock mechanism (Figure 2B). The relevance of D54 was already evident in the earlier experimental data but could only partially be explained by the protein structure of the ZF0 monomer previously predicted, as the mutation of K77 did not degrade the formation of complexes completely [14]. Hence, we believe at least dimers are still formed.

Mutations within the β‐sheet region, particularly residues 54–58, may result in the formation of monomers only. Future studies could explore whether disrupting the BCL11B tetramer to a dimer renders it nonfunctional or if complete monomerization is necessary to abolish BCL11B activity.

## 3 Conclusion

The presented study provides a detailed characterization of the BCL11B zinc finger domain (ZF0) and its multimers, advancing the understanding of its structural assembly and complex stabilization mechanisms. We successfully demonstrated that the BCL11B ZF0 domain forms higher‐order complexes, predominantly tetramers, through specific interfaces identified via replica‐exchange molecular dynamics simulations and corroborated by experimental crosslinking and size exclusion chromatography. The new predicted 3D structure of the protein also allowed the mutation experiments previously obtained by a FRET‐FACS assay to be seen and reevaluated in a new light, as not all results could be clearly explained by the previous structure.

The tetramerization process involves three distinct interfaces, whereby first, a dimer is formed through the binding of two monomers at a newly identified interface across the β‐sheet (IF‐β) forming so‐called β‐dimers. Subsequently, two of these dimers assemble to form a highly symmetrical complex, thus forming the tetramer as a dimer of dimers and two additional interfaces emerge: the previously identified IF‐α and the new IF‐γ interfaces. Our findings reveal that mutations within these interfaces can disrupt the multimerization state, emphasizing the importance of hydrophobic interactions and charge‐lock mechanisms in the formation and stability of the BCL11B tetramer. However, several mutations resulted in an altered secondary structure as evident from the presented CD spectra, but still allowed the formation of β‐dimers. Whether these changes in structure are, hence, a direct result of the amino acid substitutions or caused by the disrupted multimerization of BCL11B cannot be answered, as it is likely that the formation of a stable tetrameric complex also induces and stabilizes the structural motifs in each monomer. Further research is needed to reveal the mechanisms behind the formation and degradation of the BCL11B_42–94_ tetramer, focusing especially on the zinc‐binding properties of different mutants.

While our study has shed light on the structural assembly of BCL11B as a tetramer, its precise physiological function remains elusive. Other studies have shown that zinc finger proteins often form dimers or larger oligomers to carry out their functions [20, 21, 22]. One example is the zinc finger protein ZBRK1 (zinc finger and BRCA1‐interacting protein with a KRAP domain‐1), which forms tetrameric complexes to function correctly through its C‐terminal transcriptional repression domain [23]. Similarly, the tumor suppressor protein p53 forms tetramers via its tetramerization domain near the C‐terminal end when DNA is absent [24, 25]. This multimerization is essential for p53 to act as a transcription factor [26].

## Author Contributions

**Anne Susemihl:** investigation, data curation, formal analysis, visualization, writing – original draft, methodology, conceptualization. **Norman Geist:** software, formal analysis, visualization, methodology, investigation, writing – original draft, data curation, conceptualization. **Piotr Grabarczyk:** validation, writing – original draft, methodology, investigation. **Christian A. Schmidt:** project administration, writing – review and editing, supervision, conceptualization. **Mihaela Delcea:** conceptualization, project administration, writing – review and editing, supervision. **Lukas Schulig:** conceptualization, project administration, supervision, software, investigation, methodology, writing – review and editing.

## Disclosure

Human BCL11B: Q9C0K0.

## Supporting information


**Data S1.** Supplementary Information.

## Data Availability

The data that support the findings of this study are openly available in Raw data to: Double the double: Revisiting BCL11B's multimer at https://zenodo.org/doi/10.5281/zenodo.13284127.
