# Supplementary material for: Double the Double: Revisiting BCL11B's Multimerization
Source: Proteins. 2025 Feb 20;93(7):1205–11. doi: 10.1002/prot.26811 (PMC12127727; doi:10.1002/prot.26811)
Supplement: Supplementary file 1 — Data S1. Supplementary Information. [file PROT-93-1205-s001.pdf]

Supporting Information

Double the Double: Revisiting BCL11B's Multimerization.

Anne Susemihl[1,2], Norman Geist[1], Piotr Grabarczyk[2], Christian A. Schmidt[2], Mihaela Delcea[1], and Lukas Schulig*[3]

[1]Department of Biophysical Chemistry, Institute of Biochemistry, University of Greifswald, Greifswald
[2]Department of Hematology and Oncology, Internal Medicine C, University of Greifswald, Greifswald
[3]Department of Pharmaceutical and Medicinal Chemistry, Institute of Pharmacy, University of Greifswald, Greifswald

lukas.schulig@uni-greifswald.de
+49 (0)3834 420 4817

S1 Materials and Methods

S1.1 Experimental Section

Unless stated otherwise, chemicals were purchased from Sigma (Sigma-Aldrich, Taufkirchen, Germany). All columns used on FPLC systems and the ÄKTA instruments were purchased from Cytiva (Freiburg, Germany). The ÄKTAmicro platform was bought from GE Healthcare (Freiburg, Germany).

S1.2 Protein expression and purification

The original vector for wild-type (WT) $BCL11B_{42–94}$ was purchased from VECTORBUILDER (Vectorbuiler Inc., Santa Clara, CA, USA). Incorporated restriction enzyme sites (KpnI, XhoI) were used to generate the respective mutants. The WT plasmid was replaced by restriction enzyme digested gBlocks (Integrated DNA Technologies, Leuven, Belgium) of the respective amino acid mutants. The shortest construct $BCL11B_{42–94}$ WT and its mutants were expressed as fluorescently tagged CyPet-$BCL11B_{42–94}$ as described elsewhere.[1] In brief, CyPet-$BCL11B_{42–94}$ WT and mutants were overexpressed in NiCo21 *E. coli* cells (New England Biolabs, Frankfurt am Main, Germany) in terrific broth medium with $50\,\mu g\,mL^{-1}$ ampicillin at 37 °C. Cultures were induced at $OD_{600} = 2$ with isopropyl-beta-d-thiogalactoside (IPTG) for growing at 16 °C overnight before harvesting the cultures by

centrifugation. Cell pellets were resuspended in lysis buffer containing 20 mM Hepes pH 7.4, 150 mM NaCl, and 1 mM dithiothreitol (DTT). After clarifying the lysates via centrifugation and filtration, the lysates were loaded onto HisTrap excel affinity columns equilibrated with lysis buffer installed on an ÄKTApure platform. After loading, the column was washed with lysis buffer and eluted with buffer containing 20 mM Hepes pH 7.4, 150 mM NaCl, 250 mM imidazole, 1 mM DTT. The fluorescent tag was cleaved overnight at 4 °C using Tobacco Etch Virus (TEV) protease. Separation of tag and the untagged $BCL11B_{42–94}$ domain was carried out using the same columns, capturing the His-tagged CyPet-tag. In order to ensure high purity of the BCL11B species for the crosslinking experiments, an additional gel filtration step was added.

To generate the longer constructs $BCL11B_{42–220}$ WT and $BCL11B_{42–426}$ WT, the respective plasmids were digested with restriction enzymes and were cloned into customized vectors from VECTORBUILDER including a multiple cloning site using an InFusion Snap Assembly kit (Takara Bio Inc., Göteborg, Sweden) following the manufacturer's instructions. Purification and tag cleavage were carried out the same way as the shorter variants.

S1.3 Size Exclusion Chromatography

Evaluating the multimerization state of BCL11B species was carried out on an ÄKTAmicro platform with an installed Superdex 200 Increase 3.2/300 size exclusion chromatography column. Apparent sizes were determined using the calibration curve prepared from the manufacturer's recommended protein standards.

S1.4 Crosslinking

Crosslinking of BCL11B monomers was carried out using BS(PEG)5 linkers (ThermoFisher, Darmstadt, Germany) following the manufacturer's instructions. In short, BS(PEG)5 was freshly prepared by dissolving in dry dimethyl sulfoxide (DMSO) to a final concentration of 15 mM. BCL11B species were each prepared to a final concentration of 500 $\mu g\,mL^{-1}$ in 20 mM pH 7.4, 150 mM NaCl, 1 mM DTT. Crosslinker was added to a final concentration of 1 mM to each species. Solutions were incubated for 30 min at room temperature (RT) and the reaction was stopped by adding Tris pH 8 to a concentration of 50 mM at RT for 15 min. Crosslinking products were analyzed via denaturing tricine PAGE.

S1.5 Molecular dynamics simulations

Structure ensembles for tetrameric complexes of short $BCL11B_{54–82}$ were obtained through two independent TIGER2h^{PE} simulations,[2] with temperatures ranging from 300 K to either 450 K (R1) or 600 K (R2) and 16 replicas. Protonation states were refined using *Protein Preparation Wizard* (Schrödinger Release 2023-1). The AMBER ff14SB and Zinc AMBER Force Field (ZAFF) parameters[3] were set using tLeap (AmberTools16).[4] Sodium ions were added to neutralize the system's total charge. TIP3P water molecules were added to a cubic simulation cell with side lengths of 7 nm. Hydrogen mass repartitioning was performed with ParmEd-3.1.0 to enable a 4 fs timestep.[5] All simulations were conducted using NAMD 2.14,[6] utilizing its Tcl programming language API and the Colvars module.[7] Full periodic boundary conditions and long-range electrostatics with PME were applied in a pseudo-NPT ensemble.[8] The sampling and cooling phases were set to 16 ps and 8 ps, respectively. Pressure and temperature were maintained with a Langevin piston barostat (100 fs period and 200 fs decay

time) and a Langevin thermostat (1 ps^{-1}damping coefficient). RMSD flat-bottom restraints were applied to the backbone atoms of the proteins, allowing deviations up to 0.5 Å with a force constant of 10 $\mathrm{kcal\,mol^{-1}\,Å^{-2}}$. Implicit solvent energies for exchange decisions were evaluated using the GBOBC_{II} model with OpenMM[9] under periodic boundary conditions, with non-bonded cutoffs set to half the shortest cell dimensions. The replica-exchange simulation began after 6000 steps of energy minimization.

Table S1: Overview of TIGER2h^{PE} simulations, including the total sampling time at the baseline temperature and number of exchange cycles, the average temperature change during exchanges, and the average exchange probability.

Run	Total Time @ T_{base}	$\Delta T/\chi$	$P(\chi)$
	[µs]	[K]	[%]
R1	5.7 (356018)	19.88	43
R2	4.4 (275010)	27.53	33

S1.5.1 Contact-Contact Principal Component Analysis (ccPCA)

To improve cluster analysis results, structural ensembles of complexes obtained through TIGER2h^{PE} were filtered by contacts between each monomer and the respective three others using VMD,[10] discarding structures without contact within 0.33 nm. To identify favorable tetrameric complex structures, we utilized a contact-contact principal component analysis (ccPCA). An in-house Python code loads the molecular systems and resulting structural ensembles from TIGER2h^{PE} and selects a suitable contact mesh between both proteins. Based on the number of atoms (excluding hydrogen), the minimal number of particles from the smaller protein is then used with a corresponding stride value to select the same number of particles in the other protein, and distances between all such pairs over time are collected. Because all monomers were the same size here, the contact mesh is basically constructed between all heavy atoms and between all monomers. This data is subjected to PCA and sorted onto a 2D histogram from the 1st and 2nd principal components. The joint probability distribution is converted to the Gibbs free energy as: $\Delta G = -RT \cdot ln(\frac{P}{max(P)})$ for each state, where R is the gas constant and P is the probability of a state in PCA space. Subsequently, the data is scanned for clusters within the first two principal components using the density-based OPTICS method from scikit-learn..[11]

S1.5.2 Statistical analysis of residue contacts

Contact statistics in complexes were evaluated by counting individual residue contacts between protein chains. A contact is characterized by two atoms coming closer than 0.33 nm between two selected proteins. The counts are normalized to the number of frames and represent the average fraction of contacts per residue throughout the simulation. A value larger than one means one residue binds to multiple residues at a time. Such interaction statistics can be visualized as averages over protein surfaces or displayed in contact networks to emphasize important residues and regions for the interaction.[12]

S2 Supporting Figures

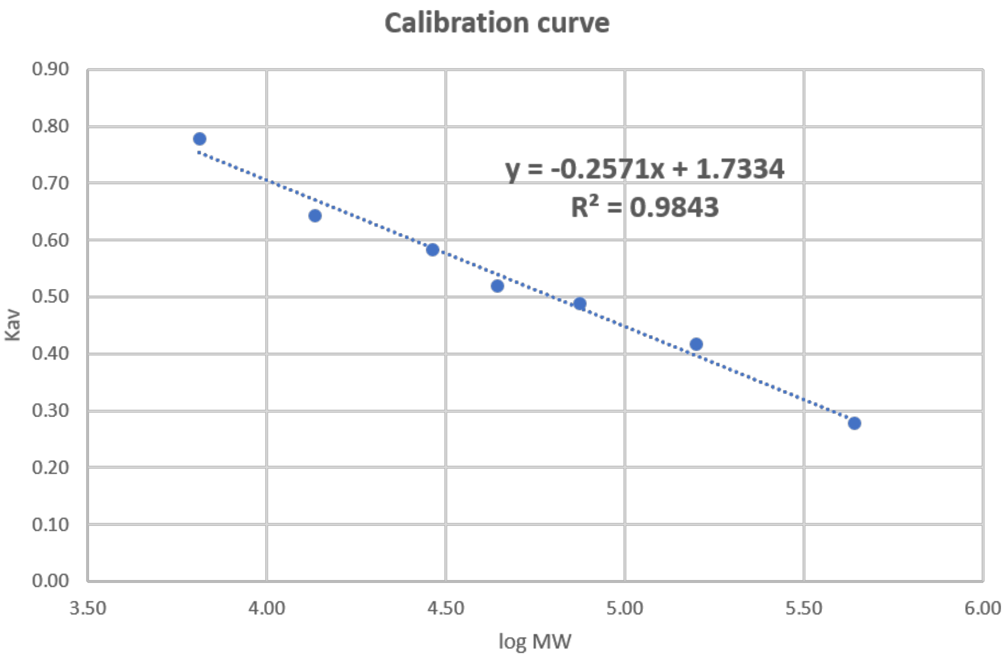

Protein	MW [Da]	log MW	K_{av}	Elution Volume [mL]
Ferritin	440000	5.64	0.28	1.41
Aldolase	158000	5.20	0.42	1.60
Conalbumin	75000	4.88	0.49	1.70
Ovalbumin	44000	4.64	0.52	1.74
Carbonic Anhydrase	29000	4.46	0.58	1.83
Ribunuclease A	13700	4.14	0.64	1.91
Aprotinin	6500	3.81	0.78	2.10

Figure S1: Calibration curve used in determining approximate sizes of the BCL11B species after analytical size exclusion chromatography. The standard curve was prepared according to the manufacturer's instructions.

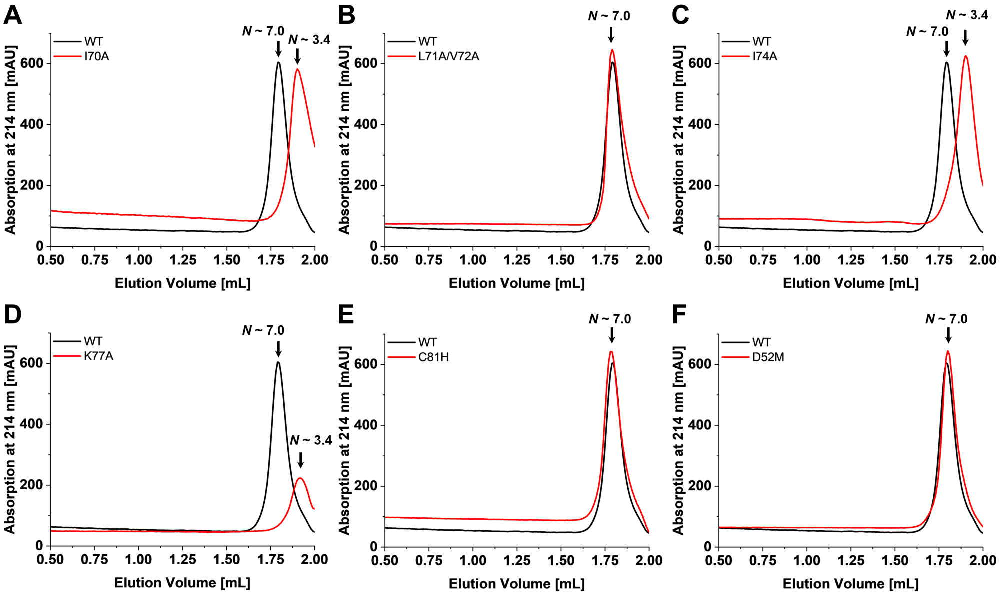

Figure S2: Size exclusion chromatography data from the small construct $\mathrm{BCL11B}_{42\text{–}94}$ WT (black) and mutants (red). Elution volume at 1.79 mL and therefore $N = 7$ roughly corresponds to calculated octamers, whereas elution at 1.9 mL with $N = 3.4$ indicates complexes of almost tetrameric size. Given the complex sizes, we observe a 2-1 ratio of size regarding WT, C81H, L71A/V72A, and D52M compared to I70A, I74A, and K77A. The small peak of K77A is due to the lower concentration of the protein, as purification of the dimeric mutant was difficult and did not result in high protein yields. Calculation of the very precise multimerization state from the molecular weight of these small constructs did not reveal suitable and crosslinking was employed instead.

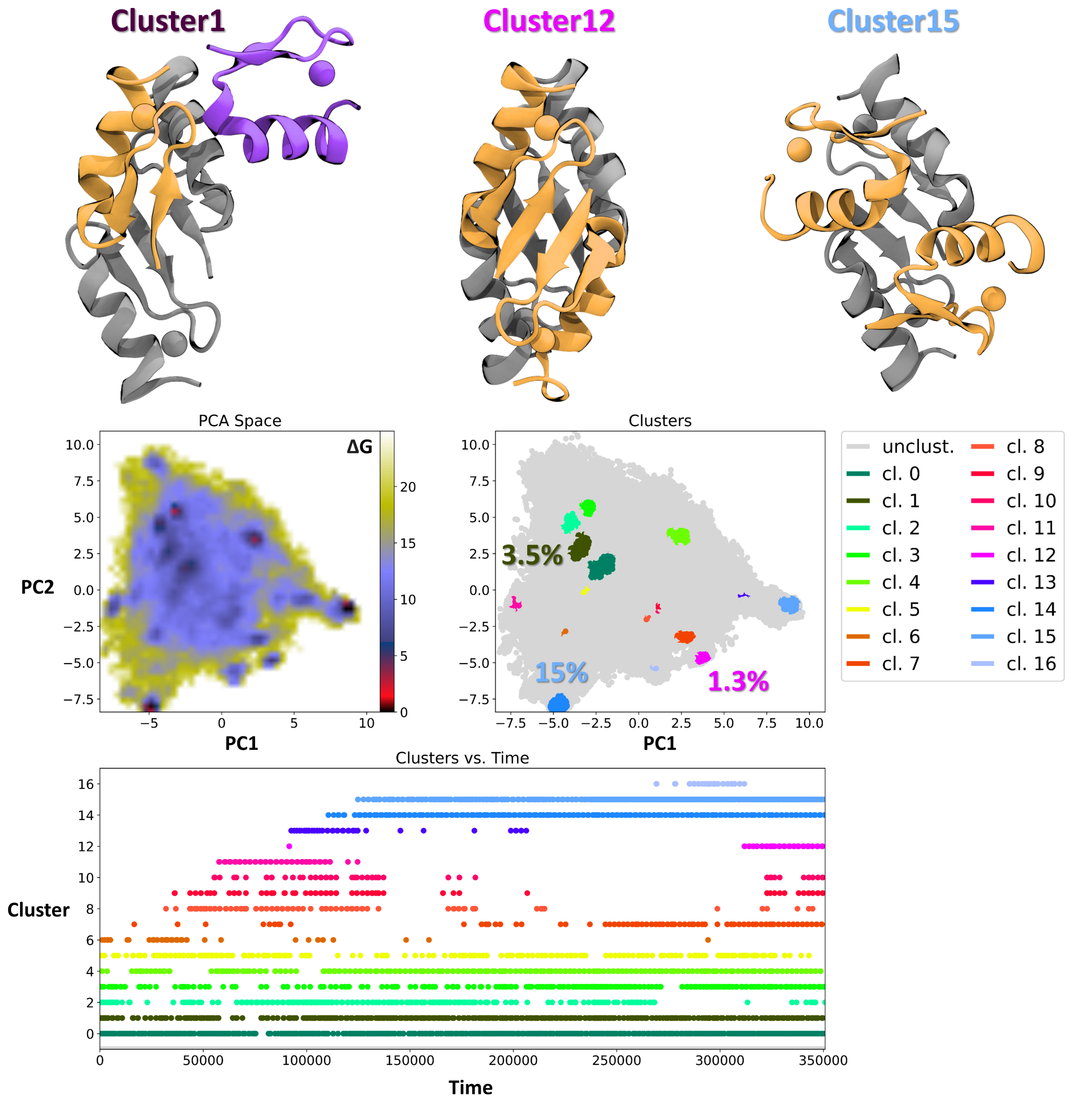

Figure S3: (top) Representative BCL11B ZF0 tetramer structures in cartoon representation as obtained from 1st TIGER2h^{PE} simulation (R1). **Cluster1** displays one example of multiple clusters in a precursor state, with three monomers assembled correctly and one remaining monomer attached to it. **Cluster12** shows the final tetramer structure, consisting of two β-dimers. The IF-α and IF-γ interfaces are only in action in tetrameric assemblies. **Cluster15** shows one of multiple larger clusters with a false tetramer, consisting of one β-dimer (gray) and one dimer across the previously found dimer interface IF-α (orange) forming a closed hydrophobic core. During this simulation, these improper tetramers were hardly overcome due to the lower maximum temperature used during sampling. (bottom) Free energy landscape (ΔG) as function of first two principal components in kcal mol^{-1} from ccPCA and resulting cluster detection.

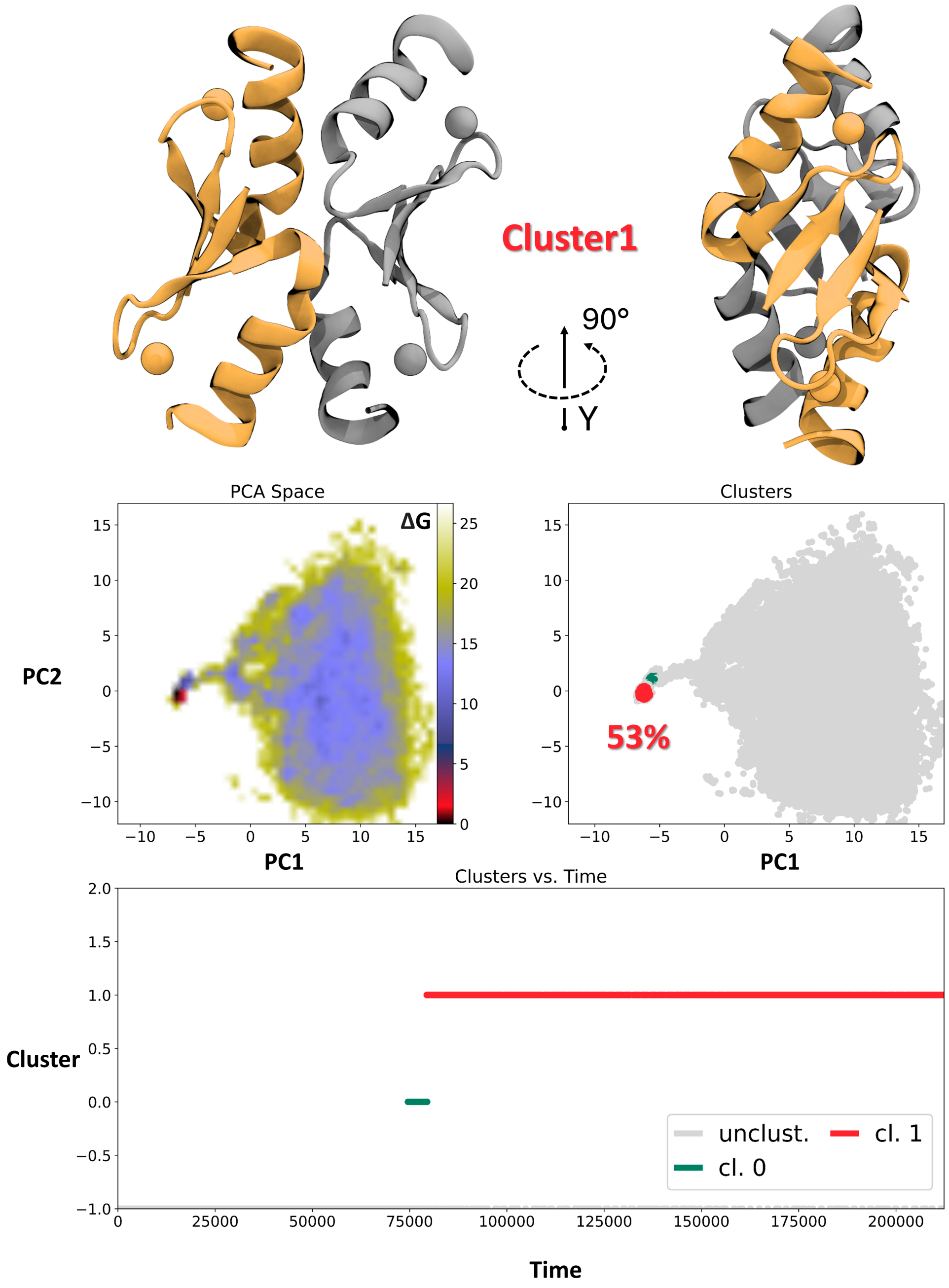

Figure S4: (top) Final BCL11B ZF0 tetramer structure in cartoon representation and different view angles as obtained from 2nd TIGER2h^{PE} simulation (R2) that used a higher maximum temperature to boost sampling. **Cluster1** shows the final tetramer structure, consisting of two β-dimers. During this simulation, once found, the final tetrameric structure dominated the conformational ensemble. (bottom) Free energy landscape (ΔG) in kcal mol^{-1} from ccPCA and resulting cluster detection.

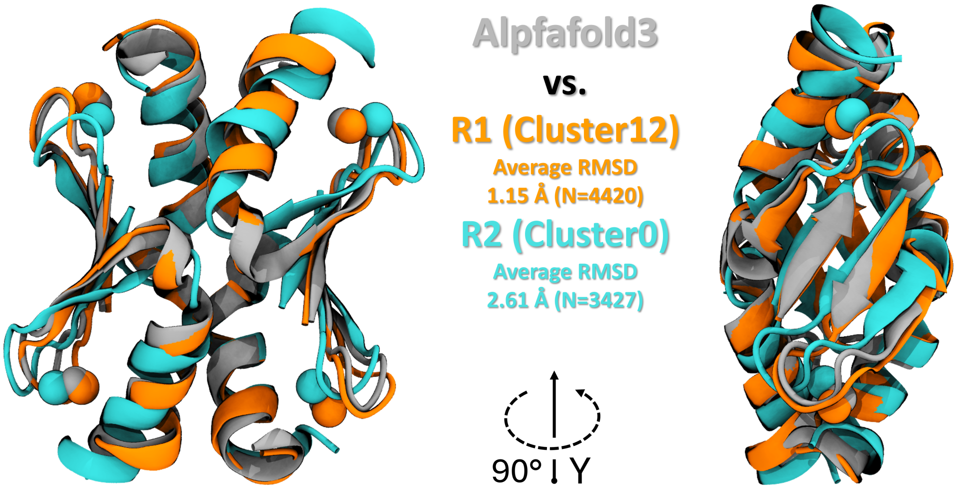

Figure S5: Comparison of tetrameric BCL11B$_{54\text{-}82}$ structures obtained through either TIGER2h^{PE} or AlphaFold 3[13] show remarkable resemblance and low average RMSD values of 1.15 Å (4420 structures) and 2.61 Å (3427) for assemblies resulting from R1 and R2, respectively.

References

[1] Anne Susemihl et al. “Easy expression and purification of fluorescent N-terminal BCL11B CCHC zinc finger domain”. en. In: *Molecules* 26.24 (Dec. 2021), p. 7576.

[2] Lukas Schulig et al. “Fundamental Redesign of the TIGER2hs Kernel to Address Severe Parameter Sensitivity”. In: *Journal of Chemical Information and Modeling* 62.17 (Aug. 2022), pp. 4200–4209. ISSN: 1549-960X.

[3] Martin B. Peters et al. “Structural Survey of Zinc-Containing Proteins and Development of the Zinc AMBER Force Field (ZAFF)”. In: *Journal of Chemical Theory and Computation* 6.9 (2010), pp. 2935–2947.

[4] David A. Case et al. “The Amber biomolecular simulation programs”. In: *Journal of Computational Chemistry* 26.16 (2005), pp. 1668–1688.

[5] Chad W. Hopkins et al. “Long-Time-Step Molecular Dynamics through Hydrogen Mass Repartitioning”. In: *Journal of Chemical Theory and Computation* 11.4 (Mar. 2015), pp. 1864–1874.

[6] James C. Phillips et al. “Scalable molecular dynamics with NAMD”. In: *Journal of Computational Chemistry* 26.16 (2005), pp. 1781–1802.

[7] Giacomo Fiorin, Michael L. Klein, and Jérôme Hénin. “Using collective variables to drive molecular dynamics simulations”. In: *Molecular Physics* 111.22-23 (2013), pp. 3345–3362.

[8] Martin Kulke et al. “Phosphorylation of Fibronectin Influences the Structural Stability of the Predicted Interchain Domain”. In: *Journal of Chemical Information and Modeling* 59.10 (Sept. 2019), pp. 4383–4392. ISSN: 1549-960X.

[9] Peter Eastman et al. “OpenMM 7: Rapid development of high performance algorithms for molecular dynamics”. In: *PLOS Computational Biology* 13.7 (July 2017), pp. 1–17.

[10] William Humphrey, Andrew Dalke, and Klaus Schulten. “VMD – Visual Molecular Dynamics”. In: *Journal of Molecular Graphics* 14 (1996), pp. 33–38.

[11] F. Pedregosa et al. “Scikit-learn: Machine Learning in Python”. In: *Journal of Machine Learning Research* 12 (2011), pp. 2825–2830.

[12] Norman Geist, Felix Nagel, and Mihaela Delcea. “Molecular interplay of ADAMTS13-MDTCS and von willebrand Factor-A2: deepened insights from extensive atomistic simulations”. In: *Journal of Biomolecular Structure and Dynamics* 41.17 (Oct. 2022), pp. 8201–8214. ISSN: 1538-0254.

[13] Josh Abramson et al. “Accurate structure prediction of biomolecular interactions with AlphaFold 3”. In: *Nature* 630.8016 (May 2024), pp. 493–500. ISSN: 1476-4687.
